# Complete mitochondrial genomes of two *Pleuronectid* species: *Clidoderma asperrimum* and *Verasper variegatus* (Teleostei: Pleuronectiformes: Pleuronectidae)

**DOI:** 10.1080/23802359.2019.1687343

**Published:** 2019-11-12

**Authors:** Han Kyu Lim, Hyo Sun Jung, Moongeun Yoon, Sang-Hwa Lee, Dong Soo Kim

**Affiliations:** a Department of Marine and Fisheries Resources, Mokpo National University, Muan Jeonnam, Republic of Korea;; b Biotechnology Research Division, National Institute of Fisheries Science, Busan, Republic of Korea;; c National Marine Biodiversity Institute of Korea, Seocheon, Republic of Korea;; d Institute of Marine Living Modified Organisms (iMLMO), Pukyong National University, Busan, Republic of Korea

**Keywords:** *Clidoderma asperrimum*, *Verasper variegatus*, mitochondrial genome, phylogeny

## Abstract

Complete mitochondrial genomes of two *Pleuronectid* species, *Clidoderma asperrimum* and *Verasper variegatus* (Teleostei: Pleuronectiformes: Pleuronectidae) were analysed using the primer walking method. Their mitogenomes were 17,632 and 17,273 bp in total length, respectively and comprised 13 protein-coding genes, 2 ribosomal RNA genes, and 22 transfer RNA genes. Their gene contents and orders were similar to those of typical vertebrates. All *Pleuronectid* species were subdivided into three clades in the phylogenetic tree, and the two *Pleuronectid* species analysed in this study formed a strong monophyletic group comprising species belonging to three genera, *Hippoglossus*, *Reinhardtius*, and *Verasper*.

Flatfishes belonging to the order Pleuronectiformes (Teleostei) are distributed worldwide. They are adapted to dwelling on the sea bottom, with conspicuous morphological characteristics including asymmetric and protrusible eyes, and dorsal fin extension corresponding to the adult stage with similar head orientation. Among pleuronectiform families, the Pleuronectidae, also known as right-eyed flounders, include commercially important species for fishing and breeding. In this study, we analyzed the complete mitochondrial genomes of two *Pleuronectid* species, *Clidoderma asperrimum* and *Verasper variegatus* (Teleostei: Pleuronectiformes: Pleuronectidae) and reconstructed a phylogenetic tree to reveal their relationship among flatfishes.

The specimens of *C. asperrimum* (PK-20182030125, 37° 2′41.91″N, 129°26′36.15″E) and *V. variegatus* (PK-20183012007, 34°20′34.37″N, 127°53′32.17″E) were collected at Uljin and Yeosu in Korea, respectively and deposited at the herbarium of Pukyong National University, Busan, Republic of Korea. Two DNA libraries were prepared using TruSeq Nano DNA LT Sample Prep Kit (Illumina, San Diego, CA, USA) from their genomic DNA followed by paired-end sequencing via MiSeq (Illumina). After trimming adapters and removing chimeras, short- and low-quality reads using cutadapt version 1.9 (Martin 2011), high-quality 22.73 and 22.28 M reads, respectively, were used for subsequent de novo assembly using Geneious version 11.1.3 (Auckland, New Zealand). Single, complete contigs were annotated with reference to MitoFish database (Sato et al. 2018).

The mitogenomes of *C. asperrimum* (MK210570) and *V. variegatus* (MK210571) were 17,632 and 17,273 bp in total length, respectively, comprising 13 protein-coding genes, 2 rRNA genes, and 22 tRNA genes. Their gene content and orders were congruent with those of typical vertebrates.

Mitogenome sequences of all *Pleuronectid* species were retrieved from GenBank. They were aligned with the *C. asperrimum* and *V. variegatus* sequences and refined manually to correct obvious misalignments. The nucleotide matrix of 12 protein-coding genes, excluding *nad6*, was created with the first, second and third bases of each codon. In addition, the unambiguously aligned regions of rRNA and tRNA genes were included in the nucleotide sequence matrix, which was partitioned into four regions based on evolutionary history (Saitoh et al. 2006): first and second codons (each 3612 bp) of protein-coding genes, rRNA genes (2703 bp), and tRNA genes (1536 bp). Two paralichthyid species were used as outgroups for the phylogenetic analyses conducted *via* RAxML version 7.0.4 (Stamatakis 2006) for maximum likelihood (ML) analysis.

Following their complete mitogenomic sequence analysis, a phylogenetic tree was reconstructed using the ML method, based on the nucleotide sequence matrix derived from 12 concatenated protein-coding genes and 2 structural RNA genes (Figure 1). The phylogenetic tree of *Pleuronectid* species included three clades. The two *Pleuronectid* species analyzed in this study formed a strong monophyletic group with species belonging to three genera: *Hippoglossus*, *Reinhardtius*, and *Verasper*. *Clidoderma asperrimum* emerged at the most basal position and *V. variegatus* clustered with its congeneric species.

**Figure 1.**
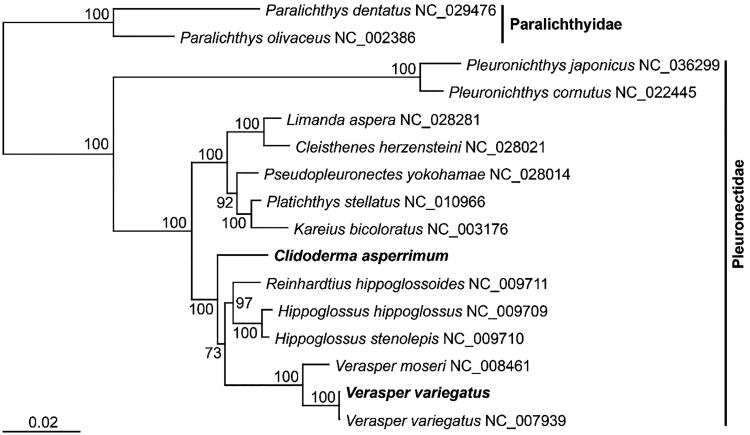
Maximum-likelihood (ML) tree inferred from the mitogenomic sequences of the species belonging to the family Pleuronectidae. The sequence matrix used in the phylogenetic analyses consisted of unambiguously aligned regions of the first and second codon positions of the protein-coding genes and ribosomal and transfer RNA genes. Bootstrap values above 50% in the ML analysis are indicated at each node. *Clidoderma asperrimum* and *Verasper variegatus* investigated in this study are shown in bold.

In this study, we provide basic phylogenetic data based on the complete mitogenomes of *C. asperrimum* and *V. variegatus* and reconstruction of the phylogenetic tree of right-eyed flounders to facilitate their breeding program.
